# Neuroscience illuminating the influence of auditory or phonological intervention on language-related deficits

**DOI:** 10.3389/fpsyg.2015.00137

**Published:** 2015-02-17

**Authors:** Sari Ylinen, Teija Kujala

**Affiliations:** ^1^Cognitive Brain Research Unit, Institute of Behavioural Sciences, University of HelsinkiHelsinki, Finland; ^2^Cicero Learning, University of HelsinkiHelsinki, Finland

**Keywords:** language deficit, dyslexia, neuroscience, training, remediation, intervention

## Abstract

Remediation programs for language-related learning deficits are urgently needed to enable equal opportunities in education. To meet this need, different training and intervention programs have been developed. Here we review, from an educational perspective, studies that have explored the neural basis of behavioral changes induced by auditory or phonological training in dyslexia, specific language impairment (SLI), and language-learning impairment (LLI). Training has been shown to induce plastic changes in deficient neural networks. In dyslexia, these include, most consistently, increased or normalized activation of previously hypoactive inferior frontal and occipito-temporal areas. In SLI and LLI, studies have shown the strengthening of previously weak auditory brain responses as a result of training. The combination of behavioral and brain measures of remedial gains has potential to increase the understanding of the causes of language-related deficits, which may help to target remedial interventions more accurately to the core problem.

## INTRODUCTION

Finding the most effective techniques to remediate language-related impairments, such as dyslexia, specific language impairment (SLI), or language-learning impairment (LLI, cf. Tallal, 2001), would be of crucial importance to educators, who try to help children struggling with these learning difficulties. This raises a question, whether understanding the neurobiological underpinnings of language impairments facilitates their efficient treatment. In this review, we discuss how neuroscience illuminates the effects of auditory or phonological intervention on dyslexia, SLI, and LLI. We focus on auditory or phonological interventions, because in many cases dyslexia, SLI, and LLI are all characterized by phonological (or auditory) deficits (Tallal, 2001; Shaywitz and Shaywitz, 2005; Pennington and Bishop, 2009; Ramus et al., 2013), despite their complex etiology. Whereas detailed brain areas influenced by reading interventions can be found in a recent meta-analysis by Barquero et al. (2014), here we address whether neuroscientific research on the remediation of language-related deficits is useful for educators and whether it has something to add over behavioral research from an educational perspective.

In the current review, the selection of publications was based on the following criteria: the research should concern dyslexia, SLI, or LLI, include testing before and after an auditory or phonological intervention or training, involve brain research measures [(functional) magnetic resonance imaging (MRI/fMRI), magnetic source imaging (MSI) or magnetoencephalography (MEG), event-related potentials (ERP), or electroencephalography (EEG)], and compare two or more groups of participants to control for the effects of repeated testing and maturation (McArthur, 2009). Searches from Web of Science and PubMed (keywords dyslexia/SLI/LLI, intervention/remediation/training, fMRI/MEG/ERP) were used in finding literature. Additional publications were found in the reference lists of relevant studies.

## IS NEUROSCIENTIFIC RESEARCH USEFUL FOR EDUCATORS?

Research on remedial interventions for learning deficits may have important applicability to education (Tallal, 2012). In this area, collaboration between education and neuroscience could result in mutual benefits (Sigman et al., 2014). However, the value of the neuroscientific approach in such research has been questioned by Bishop (2013) because of methodological and interpretive reasons. She argued that neuroscientific studies often use small subject groups, which may decrease their reliability and result in small statistical power (cf. Button et al., 2013). Furthermore, Bishop (2013) noted that some studies lack an adequate control group, which is important to control for the effects of repeated testing and maturation (see also McArthur, 2009). Indeed, future intervention studies should not only aim at having larger subject groups (Bishop, 2013) and adequate control groups (McArthur, 2009; Bishop, 2013), but also control for placebo effects (Boot et al., 2013).

Bishop (2013) also argued that the critical test of the effectiveness of interventions is the change of behavior rather than that of brain function; changes in the brain should not be considered more important than changes in behavior. However, rather than emphasizing the brain over behavior, neuroscientific intervention studies typically aim to determine the links between brain function and behavior. Importantly, understanding the link or correlation between brain activation and skills as a result of training may help to explain how and why remedial gains take place. Since the combination of neuroscientific and behavioral measures has been shown to be a better predictor of reading skills than behavioral measures alone (Hoeft et al., 2007; Maurer et al., 2009), this combination has potential to outperform mere behavioral measures in the study of remedial gains. Cognitive neuroscience has, in our opinion, also some advantages over behavioral research that were not mentioned by Bishop (2013). Especially when working with children whose motivation and skills can affect their performance considerably, a possibility to study the effects of intervention without subject’s active effort or attention is a clear advantage. This is possible, for example, by recording mismatch negativity (MMN) brain response (Näätänen et al., 2007; Kujala and Näätänen, 2010).

From educators’ perspective, neuroscientific research is seldom directly applicable in the assessment of remedial interventions. Importantly, however, educators may benefit from neuroscientific research by obtaining a more detailed picture of relevant processes underlying behavior. For example, brain measures may help to disentangle whether behaviorally observed improvement is due to the normalization of the core deficit or some compensatory strategy (e.g., Eden et al., 2004; Shaywitz et al., 2004), which is not evident in behavioral data. If, hypothetically, some intervention resulted in the formation of a compensatory function to solve some task, it may improve behavior to a certain degree but might not compete in effectiveness with the optimal function for solving that task. Still, in a large subject group, this compensatory improvement in behavior may be taken to reflect a successful intervention, if statistically significant improvement is achieved. Thus, neuroscientific research can potentially give some valuable information to educators about the deficits, which may help to target the contents of interventions more accurately.

## OVERVIEW OF STUDIES ON NEUROBIOLOGICAL CHANGES FOLLOWING PHONOLOGICAL OR AUDITORY INTERVENTIONS

As shown by **Tables 1** and **2** the majority of studies on phonological or auditory interventions focused on dyslexia or related problems in reading, writing, or spelling. Furthermore, the majority of studies have focused on children. Older age groups should not be neglected in remediation and its research, however: as noted by Eden et al. (2004), most dyslexics are adults, who may suffer from the socio-economic consequences of their reading deficit. There seem to be no constraints with respect to brain plasticity that would hinder remediation in adults or older children (Simos et al., 2002; Eden et al., 2004). Nevertheless, the earlier the interventions are conducted, the more benefit to individuals is gained, because learning is cumulative. The early gains may help to prevent difficulties not only in academic but socio-emotional domain. The optimal timing of intervention is, however, determined by maturity and acquired skills. For example, if a new skill is scaffolded by previous skills, it cannot be adapted before they are mastered (cf. Jolles and Crone, 2012).

**Table 1:** Publications including neuroscientific research on phonological or auditory remediation of dyslexia (or its risk).

Reference	Age of participants (years; mean or range)	Participant *N* (treatment; control)	Impairment or problem	Content of training	Duration of training	Brain research method	Task in testing	Behavioral improvement (pre-test vs. post-test)	Normalization of brain activation
Aylward et al. (2003)	11	10; 11	Dyslexia	Linguistic awareness, alphabetic principle, fluency, reading comprehension	2 weeks (28 h)	fMRI	Phoneme mapping, morpheme mapping	Yes	Yes
Eden et al. (2004)	41–44	19; 19	Dyslexia	Sound awareness, establishment of the rules for letter-sound organization, sensory stimulation, articulatory feedback	8 weeks (112 h)	fMRI	Repeating words, sound deletion	Yes	Yes
Gaab et al. (2007)	10	22; 23	Dyslexia	FastForWord*	8 weeks (about 67 h)	fMRI	Pitch discrimination	Yes	Yes
Hasko et al. (2014)	8	28 (11 improvers, 17 non-improvers); 25	Dyslexia	Phoneme discrimination and orthographic knowledge; phonics training	6 months (30 h)	ERP	Phonological lexical decision	Yes (improvers); no (non-improvers)	Yes (improvers); no (non-improvers)
Heim et al. (2014)	8–11	35 (12 training phonology, 7 training attention; 14 training reading); 10	Dyslexia	Phonological (Würzburger Trainingsprogramm, Kieler Leseaufbau), attentional (CogniPlus, Celeco), reading (Blitzschnelle Worterkennung)	4 weeks (10 h)	fMRI	Reading	Yes	Yes
Jucla et al. (2010)	9–11	24; 10	Dyslexia	Phonological training; visual and orthographic training	2 months (about 16 h)	ERP	Visual lexical decision	Yes (but also in controls)	Mixed (treatment group showed a different pattern than controls)
Keller and Just (2009)	8–10	35 treated poor readers; 12 non-treated poor readers; 25 non-treated good readers	Poor reading	Corrective Reading, Wilson Reading, Spell Read Phonological Auditory Training, Failure Free Reading	6 months (100 h)	DTI	–	Yes	Yes
Kujala et al. (2001)	7	24; 24	Dyslexia	Non-linguistic audiovisual matching	7 weeks (about 3 h)	ERP	Passive listening, attention directed elsewhere	Yes	Yes
Lovio et al. (2012)	6–7	10; 10	Difficulties in reading-related skills	GraphoGame: letter–sound correspondences (vs. number-knowledge game for controls)	3 weeks (3 h)	ERP	Passive listening, attention directed elsewhere	Yes	Yes
Meyler et al. (2008)	10	23; 12	Poor reading	Corrective Reading, Wilson Reading, Spell Read Phonological Auditory Training, Failure Free Reading	6 months (100 h)	fMRI	Sentence comprehension	Yes	Yes
Richards et al. (2000)	10–13	8; 7	Dyslexia	Phonological and morphological reading instruction	3 weeks (30 h)	Proton MR spectroscopy	Phonological and lexical access and a non-linguistic tone task	Yes	Yes
Richards et al. (2002)	9–12	10; 8	Dyslexia	Phonological vs. morphological reading instruction	3 weeks (30 h)	Proton MR spectroscopy	Phonological and lexical tasks, passive listening	Yes	Yes
Shaywitz et al. (2004)	6–9	37 (experimental intervention); 12 (community intervention); 28 (control)	Reading disability	Phonological intervention: sound–symbol associations, phoneme analysis, timed reading, oral story reading, dictation (vs. community intervention in school)	8 months (50 min/day)	fMRI	Cross-modal letter identification	Yes (experimental group); no (community intervention)	Yes (experimental group); no (community intervention)
Simos et al. (2002)	7–17	8; 8	Dyslexia	Phono-Graphix (phonological processing and decoding), Lindamood Phonemic Sequencing	2 months (80 h)	MSI	Pseudoword rhyme-matching	Yes	Yes
Stevens et al. (2013)	5	8; 6	Risk for reading disability	Early Reading Intervention (phonemic awareness, alphabetic understanding, letter writing, word reading, spelling, sentence reading)	8 weeks (20 h)	ERP	Selective auditory attention	Yes	Yes
Temple et al. (2003)	8–12	20; 12	Dyslexia	FastForWord*	8 weeks (about 47 h)	fMRI	Rhyme letters, match letters, match lines	Yes	Yes

DTI, diffusion tensor imaging; ERP, event-related potential; fMRI, functional magnetic resonance imaging; MR, magnetic resonance; MSI, magnetic source imaging. *FastForWord includes auditory discrimination, phoneme discrimination, phoneme identification, phonic match, phonic word, understanding instructions, grammatical structures and rules.

**Table 2:** Publications including neuroscientific research on phonological or auditory remediation of specific language impairment (SLI) or language-learning impairment (LLI).

Reference	Age	Participant *N* (treatment; control)	Impairment or problem	Content of training	Duration of training	Brain research method	Task in testing	Behavioral improvement (pre-test vs. post-test)	Normalization of brain activation
Hayes et al. (2003)	8–12	27 treated; 15 non-treated; 7 non-treated controls	Learning problems, auditory perceptual deficit	Earobics: phonological awareness, auditory processing, language processing	8 weeks	ABR, ERP	Passive listening, attention directed elsewhere	Yes	ERP yes; ABR no
Heim et al. (2013)	6–9	21; 12	LLI	FastForWord*	1 month	EEG oscillations	Passive listening and active target detection	Yes	Yes (but not all aspects)
Pihko et al. (2007)	6–7	9 (phonological intervention); 9 (physical exercise)	SLI	Speech and articulation, phoneme discrimination, phonological and linguistic awareness, rapid processing	8 weeks	MEG	Passive listening, attention directed elsewhere	Yes	Yes
Stevens et al. (2008)	6–8	8 treated SLI; 12 treated controls; 13 non-treated controls	SLI	FastForWord*	6 weeks	ERP	Auditory selective attention	Yes	Yes

ABR, auditory brainstem response; EEG, electroencephalography; ERP, event-related potential; MEG, magnetoencephalography. *FastForWord includes auditory discrimination, phoneme discrimination, phoneme identification, phonic match, phonic word, understanding instructions, grammatical structures and rules.

The studies listed in **Tables 1** and **2** suggest that in addition to behavior, the remedial gains of phonological or auditory interventions are consistently reflected in different aspects of brain functioning. These include increased or normalized brain activation as a result of training in previously hypoactive areas as measured with fMRI (Aylward et al., 2003; Temple et al., 2003; Eden et al., 2004; Shaywitz et al., 2004; Gaab et al., 2007; Meyler et al., 2008; Heim et al., 2014) and MSI or MEG (Simos et al., 2002; Pihko et al., 2007) during different cognitive tasks. MRI-based proton MR spectroscopy has shown normalized metabolism in certain brain areas after interventions (Richards et al., 2000, 2002). Training-induced changes in strength and timing of neural responses to stimulation have been demonstrated with ERPs (Kujala et al., 2001; Hayes et al., 2003; Stevens et al., 2008, 2013; Jucla et al., 2010; Lovio et al., 2012; Hasko et al., 2014). Also the time-frequency analysis of EEG has revealed amplitude increases in the oscillatory brain activity after training (Heim et al., 2013). In addition to brain function, interventions have been found to change brain anatomy, such as white matter integrity (Keller and Just, 2009). **Tables 1** and **2** also show that remedial gains, if any, consistently manifest in both behavioral and brain measures: in 16 out of 17 studies of **Table 1** and in all four studies of **Table 2**, remedial gains were found in both brain activation and skills targeted by intervention (note that Jucla et al., 2010, failed to find different behavioral improvement and similar brain response patterns between their treatment group and controls). The strong coupling of training gains in behavior and brain activation suggests that most likely the observed changes in the brain drive the changes in the behavior. As neuroscientific research may reveal the neural dynamics of processes related to behavioral performance and allows localize the deficient brain functions, it may enable to specify the neural mechanisms underlying language-related impairments and to determine brain functions and areas altered by interventions, which surface in behavior as improved skills. However, it is noteworthy that **Tables 1** and **2** lists published studies, whereas studies failing to find changes in behavior or brain activation may remain unpublished. This may cause bias toward systematically finding the coupling between neural and behavioral gains.

A recent meta-analysis of neuroscientific research exploring reading networks in the brain has suggested that dyslexia is characterized by the dysfunction of left occipito-temporal cortex, left inferior frontal gyrus, and the inferior parietal lobule (Richlan, 2012; see also Richlan et al., 2011). These brain areas are involved in phonological encoding, phonological representations, and attention, respectively (Richlan, 2012). Barquero et al.’s (2014) meta-analysis of the neuroimaging of reading interventions, in turn, suggests intervention-induced functional changes in the left thalamus, left middle occipital gyri, bilateral inferior frontal gyri, right insula, and right posterior cingulate gyrus. Thus, both Richlan’s (2012) and Barquero et al.’s (2014) findings point toward the central role of inferior frontal and occipito-temporal/occipital dysfunction in dyslexia. Correspondingly, the neuroscientific dyslexia studies included in **Table 1**, involving auditory or phonological intervention, have shown normalized brain activation, metabolism, or anatomy as a result of interventions in the occipito-temporal (Aylward et al., 2003; Heim et al., 2014) and inferior frontal (Richards et al., 2000, 2002; Aylward et al., 2003; Shaywitz et al., 2004; Heim et al., 2014) areas. In addition, normalized activation following interventions has been repeatedly observed in inferior parietal (Temple et al., 2003; Eden et al., 2004; Meyler et al., 2008, see also Richlan, 2012), superior parietal (Aylward et al., 2003; Eden et al., 2004; Meyler et al., 2008), and temporal (Simos et al., 2002; Aylward et al., 2003; Temple et al., 2003; Shaywitz et al., 2004) areas. Although inferior frontal and occipito-temporal/occipital dysfunctions, linked with phonological representations and processes (Richlan et al., 2011; Richlan, 2012), seem to be the robustest effects in dyslexia, the effects in the other areas need not to be spurious. The fact that studies use different training techniques and experimental tasks in the scanner during neuroimaging may account for finding remedial changes in different brain functions and areas (Heim et al., 2014).

Neuroimaging research on the effects of auditory and phonological intervention is complemented by ERPs, reflecting the dynamics of neural responses. Studies on dyslexia (**Table 1**) have shown that treatment strengthens brain responses, such as MMN (Kujala et al., 2001; Lovio et al., 2012), attention-related ERP (Stevens et al., 2013), and N400 (Hasko et al., 2014). Lovio et al. (2012) observed also a treatment-induced shortening of the MMN latency across groups receiving grapheme-phoneme or number-knowledge training. In SLI and LLI (**Table 2**), the remedial gains of interventions have been observed as the strengthening of oscillatory brain activity (Heim et al., 2013) and auditory cortical responses, including MMN (Pihko et al., 2007) and attention-related ERPs (Stevens et al., 2013), which are accompanied by improved performance in behavioral language tasks. Training has also been shown to shorten the latency of auditory P1-N2 complex, resulting in a more mature response pattern (Hayes et al., 2003). As the MMN study by Pihko et al. (2007) involved passive listening, where participants’ attention was directed elsewhere, enhanced MMN responses indicate remedial effects on low-level, pre-attentive auditory processing that is modified by phonetic representations.

## DOES THE CONTENT OF THE INTERVENTION MATTER?

From educators’ perspective, it would be important to conduct interventions that tap the core deficit rather than induce compensatory improvements. Direct comparisons using the same experimental tasks but different interventions could clarify, whether the optimal method of remediating language-related deficits can be found. To this end, Shaywitz et al. (2004) have compared the effects of a targeted experimental intervention and a community intervention on behavioral performance and brain activation in children with reading difficulties. The experimental intervention focused specifically on phonological skills with different kinds of tasks, whereas community intervention consisted of activities commonly provided in school, such as remedial reading (see Shaywitz et al., 2004, for details). As a result of interventions, the experimental intervention group had achieved significant gains in reading fluency and showed an increased activation of left-hemisphere brain regions, whereas no such gains were observed after community intervention. This result emphasizes that the nature of intervention is critical for its success (however, see Boot et al., 2013, for discussion on the expectations about improvement).

Besides showing a correspondence between improvements in skills and changes in neural function, neuroscientific measures can illuminate the specific effects of interventions on brain areas subserving distinct cognitive functions. Heim et al. (2014) compared three different kinds of training that focused on phonology, attention, and visual word recognition (reading). They divided school-aged dyslexic children into three training groups according to their cognitive profiles. All training methods improved children’s reading skills to a similar degree. During a reading task in an fMRI scanner, all training programs resulted in the increased activation of the visual word form area, located in the left fusiform gyrus. In some other brain areas, however, the training programs had different effects on brain activation: phonological and reading training increased activation in bilateral parietal areas, whereas attention training increased activation in the left temporal cortex. Thus, different training programs had both shared and specific effects on brain activation, which would not have been evident on the basis of behavior alone.

In line with Heim et al.’s (2014) conclusions on shared effects induced by different training types, very different kinds of interventions have resulted in significant behavioral and neural gains in individuals with language-related deficits. For example, many studies (see **Tables 1** and **2**) have shown the remedial gains of phonological training with FastForWord, including auditory discrimination, phoneme discrimination, phoneme identification, phonic match, phonic word, understanding instructions, and grammatical structures and rules. Significant remedial gains in brain activation and behavior have, however, been obtained also with non-linguistic tasks that, at first sight, might seem to have a less obvious link to language-related deficits. Kujala et al. (2001) presented dyslexics with an intervention with non-linguistic audiovisual training, including matching a sequence of non-speech sounds with a sequence of visual shapes. As a result of intervention, reading accuracy had improved and MMN brain responses to tone-order reversals had increased. The change in reading skills and MMN amplitude significantly correlated, suggesting an association between reading abilities and non-linguistic processing. In a similar vein, Gaab et al. (2007) used non-speech stimuli with rapid transitions to remediate dyslexia. After training, language and reading skills had improved and prefrontal regions associated with the processing of rapid transitions were more strongly activated than before training. The remedial gains for reading skills from very different kinds of intervention tasks allude to the possibility that they tap some common, domain-general process involved in, and perhaps necessary for, reading and language skills and contribute to remedial gains along with domain-specific effects.

A candidate function that may, in concert with others, participate in domain-general remedial gains is attention. Stevens et al. (2008) studied whether linguistic intervention would improve selective attention in children with SLI. As a result of training, measures of receptive language had improved and previously attenuated event-related brain responses reflecting selective attention had normalized. Stevens et al. (2013) have also suggested that children at risk for reading difficulty show atypical brain measures of selective attention, which can be remediated by reading intervention. These findings suggest that language skills and auditory attention are strongly connected, complementing the earlier findings on the role of visual attention in dyslexia (Facoetti et al., 2000; Valdois et al., 2004; Shaywitz and Shaywitz, 2008; Vidyasagar and Pammer, 2010; Franceschini et al., 2012; Vogel et al., 2012). This is in line with models of dyslexia proposing the dysfunction of the inferior parietal lobule, which has been linked to attention (Richlan, 2012), and the observations of normalized training-induced activation in the inferior parietal areas (Temple et al., 2003; Eden et al., 2004; Meyler et al., 2008).

## HOW LONG-LASTING ARE THE REMEDIAL GAINS IN THE BRAIN?

Interventions aim at long-lasting gains. The dynamics of neural changes induced by intervention can be explored with follow-up neuroimaging studies, which enable to specify brain functions that show long-term effects. Shaywitz et al.’s (2004) experimental intervention group of dyslexic children returned to an fMRI scan 1 year after the intervention. The normalization of activation pattern was seen both immediately after intervention as well as 1 year after it, suggesting long-lasting remedial effects. Similarly, Meyler et al. (2008) observed hypoactivation of parietal areas before intervention in poor readers and increased activation of these areas immediately after intervention. Interestingly, when they conducted a follow-up 1 year after the intervention, they found that the activation of the parietal areas had continued to increase. Thus, the activation pattern of previously hypoactive areas had normalized, probably reflecting cumulative learning effects following intervention. These follow-up studies thus show that treatment-induced neurobiological changes, coupled with improvement in behavioral performance, can be long-lasting and may enable cumulative gains in language-related skills.

## CONCLUSION

Interventions and training programs involving phonological and auditory tasks have repeatedly gained remedial effects in dyslexia, SLI, and LLI. Neuroscientific research has demonstrated that improved behavioral performance is coupled with changes in both brain function and brain anatomy. Neuroimaging has revealed normalized training-induced brain activation patterns, whereas electrophysiological measures have demonstrated the normalization of strength and timing of brain responses and oscillatory activity after training. Training effects have been observed also in white matter. Especially in the study of dyslexia, neuroscientific studies have illuminated the location of aberrant brain functions, which has enabled to specify the models of the impairment. Neuroimaging studies have also highlighted partly similar and partly specific patterns of neural activation as a result of different training programs. Gains from very different phonological and auditory tasks as well as training effects in the parietal cortex support the models that propose the involvement of some domain-general neural mechanisms, such as attention, in language-related impairments.

In our opinion, neuroscientific studies thus give an important contribution to the treatment of language-related impairments. Specifically, we argue that the use of both neuroscientific and behavioral measures in intervention studies can increase the understanding of how and why interventions change the deficient neural networks, if methodological requirements are met (cf. Bishop, 2013). From educators’ perspective, neuroscientific research methods are seldom directly applicable to the assessment of remedial interventions. However, keeping up-to-date in such research can provide educators with better understanding of the causes of language-related impairments and help them to target interventions more accurately.

## Conflict of Interest Statement

The authors declare that the research was conducted in the absence of any commercial or financial relationships that could be construed as a potential conflict of interest.
